# A New Medical Record Proposal to the Prognostic Risk Assessment for MRONJ in Oncologic Patients: “Sapienza Head and Neck Unit” Proposal

**DOI:** 10.3390/ijerph18041851

**Published:** 2021-02-14

**Authors:** Edoardo Brauner, Silvia Mezi, Alessandro Ciolfi, Chiara Ciolfi, Resi Pucci, Andrea Cassoni, Andrea Battisti, Gabriele Piesco, Francesca De Felice, Nicola Pranno, Matteo Armida, Francesca De Angelis, Umberto Romeo, Mauro Capocci, Gianluca Tenore, Vincenzo Tombolini, Valentino Valentini, Livia Ottolenghi, Antonella Polimeni, Stefano Di Carlo

**Affiliations:** 1 Department of Oral and Maxillo-Facial Sciences, Sapienza University of Rome, 00161 Roma, Italy; edoardo.brauner@uniroma1.it (E.B.); chiara.ciolfi2402@gmail.com (C.C.); resi.pucci@gmail.com (R.P.); andrea.cassoni@uniroma1.it (A.C.); andrea.battisti@uniroma1.it (A.B.); nicola.pranno@uniroma1.it (N.P.); matteoarmida@virgilio.it (M.A.); francesca.deangelis@uniroma1.it (F.D.A.); umberto.romeo@uniroma1.it (U.R.); dott.mauro.capocci@gmail.com (M.C.); gianluca.tenore@uniroma1.it (G.T.); valentino.valentini@uniroma1.it (V.V.); livia.ottolenghi@uniroma1.it (L.O.); Antonella.Polimeni@uniroma1.it (A.P.); stefano.dicarlo@uniroma1.it (S.D.C.); 2 Department of Radiological Oncological and Pathological Sciences, Sapienza University of Rome, 00161 Roma, Italy; silvia.mezi@uniroma1.it (S.M.); gabriele.piesco@uniroma1.it (G.P.); francesca.defelice@uniroma1.it (F.D.F.); vincenzo.tombolini@uniroma1.it (V.T.)

**Keywords:** osteonecrosis, metastatic bone cancer, dental treatment, medical report, oral health care delivery

## Abstract

Medication-related osteonecrosis of the jaw (MRONJ) is an adverse event associated with antiresorptive and antiangiogenic drugs. The use of these drugs in the treatment of cancer patients with bone metastasis is necessary and standardized in the literature. A multidisciplinary approach for the patient’s management is strongly recommended. Therefore, it should be necessary to integrate the path of these subjects with a dedicated dental screening in order to first assess the individual risk of developing a MRONJ, and then to plan dental treatments and oral hygiene sessions, and finally to schedule a follow-up to intercept and treat early osteonecrosis. The aim of this manuscript is to propose a new simple medical report to evaluate patients affected by metastatic bone cancer in order to reduce the risk of developing MRONJ.

## 1. Introduction

The relationship between bisphosphonates and osteonecrosis of the jaw was first formulated by Marx in 2003 [1]. Bisphosphonates are a category of medication that inhibits osteoclast-mediated bone organic processes and bone transforming by caspase-mediated cell death; they are approved for the prevention and delay of the complication of skeletal connected events. Currently, the right term to refer to this condition is medication-related osteonecrosis of the jaw (MRONJ); the amendment of the signifier from BRONJ (biphosphonates-related osteonecrosis of the jaw) to MRONJ is even to elucidate the growing range of osteonecrosis cases involving the jaws related to antiangiogenic and antiresorptive medications [2,3,4]. 

The American Association of Oral and Maxillo-facial Surgeons (AAOMS) [5] states that patients are affected by MRONJ if they have all of the following characteristics: (I) present or antecedent antiresorptive or antiangiogenic treatment, (II) exposed bone or persistence of a intraoral or extraoral fistula in the maxillofacial region that allows bone probing for more than eight weeks, as well as patients with stage 0 with prodromal disease (unexposed variant) [6,7,8], and (III) no history of radiation therapy or metastatic disease to the jaws.

Different osteonecrosis’ incidence between osteoporosis and cancer patients is highlighted in the literature; more important differences have been underlined between patients taking drugs intravenously rather than orally. The inhomogeneity of the data makes it difficult to define a real and cumulative incidence of this pathology. This, however, represents a significant problem as patients who develop recurrent MRONJ infections in the oral cavity can face serious complications, such as the suspension of oncological treatment in severe cases [9].

While the incidence of BRONJ in patients taking oral bisphosphonates ranges from 1.04 to 69 per 100,000 patient-years, in contrast, patients affected by osteoporosis present an incidence of DRONJ (drug-related osteonecrosis of the jaw) ranging from 0 to 30.2 per 100,000 patient-years. In consideration of these data, the AAOMS concluded that the incidence of ONJ (osteonecrosis of the jaw) in patients with osteoporosis is low. However, the incidence of BRONJ in oncologic patients receiving intravenous bisphosphonate therapies ranges from 0 to 12,222 per 100,000 patient-years and this increases considerably if the subjects are patients with malignancies taking subcutaneous denosumab, with a range from 0 to 2316 per 100,000 patient-years. Information about the incidence of ONJ induced by angiogenesis inhibitors could not be found in a reliable literature such as systematic reviews and/or consensus statements [10].

MRONJ can be challenging to treat and can cause significant pain and reduced quality of life for patients. Because of the difficulties in treating and the incidence of this disease, a correct diagnosis and staging are appropriate. Clinicians should investigate the presence of above mentioned AAOMS’ criteria to confirm a diagnosis of MRONJ [11].

A well-established staging system should be used to quantify the severity and extent of MRONJ and to guide management decisions and treatment strategies. The 2017 International Task Force on Osteonecrosis of the Jaw staging system for MRONJ, the 2014 American Association of Oral and Maxillofacial Surgeons staging system, and the Common Terminology Criteria for Adverse Events version 5.0 are considered suitable [5]. The same system should be used by all the different clinicians that are treating MRONJ patients.

MRONJ can be initially treated with a conservative approach; this may include antimicrobial mouth rinses, antibiotics if clinically indicated, effective oral hygiene, and conservative surgical interventions [12].

Refractory MRONJ can be treated with surgical interventions: if symptoms persist despite the initial treatment and keep affecting function, an aggressive surgical approach (e.g., block resection of necrotic bone or soft tissue closure) can be adopted. However, if bone exposure is asymptomatic, this technique is not recommended. Obviously, risks and benefits of the proposed treatment should be clearly analyzed by a multidisciplinary team together with the patient.

If MRONJ is diagnosed while the patient is being treated with BMAs (bone-modifying agents), evidence is not sufficient to support or refute the discontinuation of the BMAs. Administration of the BMA is under discretion of the treating physician.

During the course of MRONJ treatment, the objective and subjective status of the lesion should be discussed by the dental specialist together with the oncologist.

Coordination of care is fundamental to decrease the risk of MRONJ. It is important to perform an oral care assessment before the therapy when a nonurgent BMA treatment is scheduled for cancer patients. Consequently, a dental care plan should be developed depending on the assessment and it should be coordinated by the dentist together with the oncologist; necessary dental procedures should be performed before starting BMA therapy. The dentist should then schedule a follow-up routine after the beginning of the BMA treatment, for example every 6 months. Moreover, modifiable risk factors including poor oral health, invasive dental procedures, ill-fitting dentures, uncontrolled diabetes mellitus, and tobacco use should be identified and treated by the multidisciplinary team, motivating the patient too [13].

The proposal of this new simple medical report is to identify the clinical risk factors for MRONJ including dental surgery, tooth extractions, poor oral hygiene, not congruous dental prostheses, and the concomitant exposure to other drugs especially angiogenesis inhibitors and corticosteroids [5,14,15]. 

At the Department of Oral and Maxillofacial Sciences of “Sapienza” University of Rome, a multidisciplinary team set up in 2007 the CROMA Project (Coordination of Research on Osteonecrosis of the Jaws) [16], whose aim is to prevent or treat established MRONJ and to give relevant information and advice both to patients and to prescribing providers. In 2015, a new project called MoMax (MedicinaOrale e Maxillofacciale—Oral and Maxillofacial Medicine) was born in order to multidisciplinarily take care of head and neck cancer patients. This task force is primarily formed by dentists and oral and maxillofacial surgeons, but several experts (otolaryngologists, oral pathologists, oncologists, radiotherapists, and an expert in statistics) often join the group to provide a comprehensive patient-centered oral care delivery. 

Starting this project raises the need to organize a dedicated medical record for these patients, in order to assign a risk class according to the patient’s clinical history and objective evaluation. According to the score assigned to the patient, the controls to perform oral hygiene and the monitoring of oral health will then be programmed so that an early diagnosis of MRONJ can be made. The personalized risk of developing a MRONJ in bone metastatic patients before starting antiresorptive and antiangiogenic agents will be assigned, and according to this assessment, suggest the most appropriate oral treatment choice and the subsequent follow-up during bone therapy, based on the previously described care pathway clinical experience [17]. Once the risk factors have been eliminated, frequent follow-up sessions are recommended to facilitate early diagnosis of pathology onset. If periodic checks are carried out, the timing of early interception of a possible pathological development of MRONJ is improved. This allows greater protection for the patient, less invasiveness of the sessions, simpler interventions for clinicians, and lower intervention costs. Inserting the risk stage of MRONJ into the healthcare delivery allows intervention in early stages, improving the outcome of these patients. In addition, with the aim of a health system such as the Italian one, an early intervention in these patients results not only in a better outcome for the patient but also in a reduction in the costs of management (hospitalization, surgery costs, and medicaments).

The dedicated clinical path should be set up as follows.

## 2. Materials and Methods

### 2.1. Dedicated Clinical Path

Oncological patients at the first diagnosis of bone metastases were discussed in a multidisciplinary board composed of a clinical oncologist, a radiation oncologist, a radiologist, and a dentist, in accordance with the directions of the AAOMS committee on MRONJ that supports a multidisciplinary approach for treatment with antiresorptive or antiangiogenic medications [5]. The oncological and bone therapy were defined; patients were classified as critical, serious, or stable depending on the severity of cancer condition and bone involvement (Table 1b). 

### 2.2. Dental Examination 

All information concerning the oncological patient’s conditions and the risk of MRONJ due to oncological and bone treatment planned (Table 1a) (score 1 to 3) must be known to oral health professionals before the dental evaluation. 

Prior to starting the patient’s oral examination, his history must be obtained and recorded in order to assess the risk of osteonecrosis. 

### 2.3. Radiographic Examination

Orthopantomography or CT Cone Beam must be made before starting bone therapy [5]. 

### 2.4. MRONJ Risk Score

The score is obtained by four different parameters:

1. pharmacological related score and treatment planned (Table 1a) (antiresorptive drugs risk = 1, antiresorptive plus antiangiogenics risk = 2, antiresorptive plus antiangiogenics plus corticosteroid risk = 3);
2. second reflects the oncological status at baseline (Table 1b);
3. the dental and oral score assigned (Table 2) (from 0 = no risk to 3 = highest risk);
4. the medical history risk factors (Table 3).

At the end of the dental evaluation, the patients may receive bone therapy. The patients must be evaluated again after 30 days of bone therapy and the check-up frequency depends on the last assigned score.

The medical records that we have developed are an effective tool to quickly identify individual risk factors related to MRONJ. The baseline dental score is awarded on the first visit, before the patient starts the pharmacological therapy. This score emerges from the assessment of dental caries (number and level), tooth mobility, periodontal disease, the presence of fragments of root caries, periapical disease, missing teeth, and stability and denture congruence or dislocations [18,19]. 

The role of the dentist is to work on the dental score in order to decrease it. The aim is to bring the initial dental score to “0” when feasible. In cases of included teeth or periodontal diseases, the score never will arrive to “0”; this gives the sense to the whole work since it is deduced that the clinical attention to the patient will never decrease.

The treatments are impossible in patients with critical or serious conditions. For these patients it is mandatory to prevent or minimize any delay to oncological treatment. The dental treatment may be even deferred after having stabilized the overall condition of patients on the basis of the degree and severity of skeletal related events (SREs) during bone therapy, but only if invasive dental treatment is excluded (score 1 or 2) (Table 3). 

However, in all cases of non-restorable and those with poor prognosis (score 3) teeth should be extracted. Dentoalveolar surgery should be performed before undergoing therapy. Oncologic therapy should not be performed before the wound is completely healed (14 to 21 days). 

In patients with total or partial removable dentures, any intraoral prosthesis is removed before starting the examination. 

Attention should be paid to the risk of mucosal trauma: any decubitus must be immediately resolved, the prosthesis should be rebased when necessary and must be replaced if it is incongruous. It is not suggested to leave the patient without a prosthesis during cancer treatment so as to restore the physiologic occlusion plane and to allow a correct masticatory and aesthetic function [20]. Patients must be educated to the importance of regular checkups and to immediately report to the dentist the occurrence of pain, swelling, or mucosal inflammation. As these are non-specific symptoms, it is necessary to instruct the patient on the need to consult the dentist of their appearance [21]. 

At this point, the score mainly depends on drugs and comorbidities, and may vary from 1 to 2 points in most cases in our experience, and the anti-resorptive and antiangiogenic treatment can be started safely. 

Depending on the individual final score obtained the patient will be entered in a follow-up program as shown in the Appendix A. On the assumption that in healthy patients (risk of MRONJ = 0), a complete dental examination and oral hygiene is normally recommended once a year and once every six months; in the case of healthy patients with fixed, removable, or implant supported prostheses, we recommend a check-up every six months in the event of a score = 1, every four months if score = 2, every three months if score = 3, every two months if score = 4, every month if score = 5 or more.

Dental prophylaxis, dental hygiene, caries control and conservative restorative dentistry must be continued indefinitely. 

## 3. Discussion

Bone health is critically important for patients with metastatic cancer. Aminobisphosphonate, Receptor Activator of Nuclear factor Kappa-Β Ligand (RANK-L), and antiangiogenic agents have reported an increase of time to first and subsequent skeletal related events SRE(s) in large phase III studies. The beginning of bone therapy should be delayed to a multidisciplinary consulting in which the risk of major side effects of SREs as hypercalcemia, pathological features of long bones, and spinal cord compression must be assessed. Furthermore, any urgent need to begin systemic therapy or local palliative treatment and pain control must be considered, and the risk factors of MRONJ must be defined. Indeed, the safety profile of aminobisphosphonate, RANK-L ligand, and antiangiogenic agents showed that the exposure to chronic treatment could cause MRONJ, a significant adverse event that has an impact on patients’ quality of life [22,23]. The risk of osteonecrosis could be minimized by the complete removal of the predisposing factors and any possible triggers in the subsequent course of bone therapy [24]. Indeed, it is estimated that MRONJ rarely occurs spontaneously, more often after dentoalveolar surgery [25,26], infection, or trauma in patients with current or previous treatment with antiresorptive or antiangiogenic agents. It is necessary to know all the available information on the clinical status of patients, as well as the presence of any risk factors of MRONJ and patient comorbidities before dental assessment to modulate the aggressiveness of dental care [27]. Therefore, the need for integration between the various professionals involved in the treatment is strongly suggested, before, during, and also after the suspension of the bone therapy. Before starting treatment with antiresorptive therapy or antiangiogenic therapy, a thorough examination of the oral cavity and a radiographic evaluation are required. It is important to identify any acute infection and potential infection sites to prevent future problems that may be difficult to manage during bone therapies [5]. 

We underline that the management of patients with periodontal disease or included teeth can be critical as their dental score is likely to remain high. In fact, in these patients the sacrifice of many teeth that also potentially could be saved in non-metastatic patients became mandatory with the delay of cancer therapy until complete wound healing and the need for frequent checking becomes mandatory to reduce the risk of MRONJ in these high-risk patients. 

Patients candidates to implant treatment need a separate mention. The goal of implant rehabilitation is to improve the quality of life of these patients [28,29], however there is no data in literature on implant treatment in cancer patients at risk of MRONJ, so the risk is considered to be associated with that of an extraction or other surgery. Thus, after weighing risks and benefits, it must be concluded that implant placement secondary to treatment with antiresorptive and antiangiogenic drugs is not indicated in cancer patients. The alternative may be to insert implants before starting bone therapy. Regarding cancer patients, however, we must consider two fundamental aspects: the urgency of cancer care and the patient’s life expectancy. It is obvious that cancer care should be started as soon as possible, and implant treatment would risk delaying therapy. In the case of bone metastases, life expectancy at 5 years varies from 5 to 40% [30] depending on the type of the tumor, the extent of disease, and its biological behavior. At this point, each of us is faced with ethical questions: to what degree is it right to subject these patients to a more or less complex rehabilitation program? Compared to life expectancy, is it right to propose a rehabilitation treatment? The decision must be made each time together with the clinical oncologist, based on the patient’s wishes, evaluating in each case pros and cons [31,32,33]. 

Patients should be kept under close observation, so during the dental examination it is extremely important to motivate the patient regarding dental care; the importance of fluoride applications and rinses with chlorhexidine (0.12% daily) must be clearly specified [34]. If despite all the attention, a surgical intervention becomes necessary (for example, a tooth extraction), the goal is to be the most conservative possible so as to minimize the trauma [32,33,34,35]. Bone manipulating should be minimal, burs should be avoided, as well as anesthetics with vasoconstrictors (which would decrease the blood supply), the local hemostasis must be accurate, the antibiotic coverage must be carried out with broad-spectrum antibiotics to be administered before and after surgical treatment for at least fifteen days. 

Post-surgical follow-up must be appropriate to ensure complete healing. Drug holidays should be considered if systemic conditions permit reducing the risk of developing MRONJ and clinical symptoms [36,37].

## 4. Conclusions

In this study, a dental clinical record is proposed to evaluate patients who have to undergo bone therapy. This folder allowed us to easily classify our patients based on the risk of MRONJ and place them in a proper plan of oral treatment and follow-up. It is based on all the parameters that may influence dental treatment decisions: patient’s general condition, timing to start bone therapy and chemotherapy, and any condition that may increase the risk of MRONJ. We strongly suggest that a dental visit with a statement of no impediment must be mandatory before starting the use of antiresorptive and antiangiogenic drugs to prevent MRONJ [38,39]. We also stress the need to inform all patients of the low statistical risk of experiencing MRONJ when following the prevention protocols, and the risk incurred by not fulfilling the dental follow-up. Finally, the complexity of protocols of prevention of MRONJ, allow us to suggest in most of the cases, especially in patients with severe periodontal disease, the emerging need to centralize patients; most of the treatment can be performed in every dental structure, private or public, however, the clinical decision for each patient at the beginning and during the antiresorptive and antiangiogenic treatment must be taken in a multidisciplinary collegiate meeting. 

## Figures and Tables

**Table 1:** (a): Pharmacological related score; Medium risk (1) antiresorptive drugs (Zometa 4 mg iv, Denosumab 120 mg sc). High risk (2) antiresorptive drugs+ angiogenesis inhibitors. Severe risk (3) antiresorptive drugs+ agiogenesis inhibitors+ corticosteroid and/or chemotherapy; (b): Oncological status at baseline and related score. Stable (0): bone disease only. No critical locations. Hormone therapy preferred. Low delay the start of treatment could be planned. Antiresorptive therapy could be delayed until dental health is optimized. Serious (1): systemic disease not life threatening, bone metastases not critical, minimize delay to definitive treatment. Antiresorptive therapy could be delayed until dental health is optimized. Critical (2): life threatening disease in visceral site or bone disease in critical locations. Prevent any delay to definitive treatment. Antiresorptive therapy could be delayed until dental health is optimized.

**(a) Pharmacological Planned Related Score (See Table 1a and complete the boxes, then tick 1, 2 or 3).**				
**Medication Related**	**Molecule**	**Antiresorptive**	**Antiresorptive + Antiangiogenic Agents**	**Antiresorptive + Antiangiogenic Agents + Corticosteroid**
	**dosage**			
	**way of administration**			
**Score**		1	2	3
**(b) Oncological status at baseline and related score (See Table 1b and tick the box).**				
**Oncological Status at Baseline**		**Stable**	**Serious**	**Critical**
		0	1	2

**Table 2:** Dental score and dental treatment.

	Score	Critical	Serious	Stable
**Denture**	0 No—mobile congruous	/	/	/
	1 Fixed on vital tooth—mobile incongruous	/-remake mobile prosthesis	/-remake mobile prosthesis	/-remake mobile prosthesis
	2 Fixed on devitalized tooth	/	/	/
	3 Fixed on damaged tooth	Denture removal + Tooth extraction	Denture removal + hopeless tooth extraction	Same protocols of a healthy patient
**Decay**	0 No	/	/	/
	1 I, III, V class	Immediate treatment	Deferrable	Deferrable
	2 II class	Immediate treatment	Deferrable	Deferrable
	3 Pulp involvement	Tooth extraction	Tooth extraction	Endodontic treatment and prosthetic restoration
**Root**	0 No	/	/	/
	1 Devitalized	Tooth extraction	Tooth extraction	Prosthetic restoration
	2 Non-devitalized	Tooth extraction	Tooth extraction	Endodontic treatment and prosthetic restoration
	3 Hopeless	Tooth extraction	Tooth extraction	Tooth extraction
**Periodontal Disease** **(Armitage and Linde 1999)**	0 No	/	/	/
	1 Low gingivitis and recession 1 mm < CAL < 2 mm	Curettage	Curettage	Curettage
	2 Modest socket < 4 mm with no mobility 3 mm < CAL< 4 mm	Curettage	Curettage	Tooth extraction
**CAL = Clinical Attachment Level**	3 Serious socket > 4 mm and/or tooth mobility CAL > 4 mm	Tooth extraction	Tooth extraction	Tooth extraction
**Biological Periimplant** **Complications**	0 No	/	/	/
	1 Gingivitis	Gingivectomy	Gingivectomy	Gingivectomy
	3 Perimplantits	Extraction	Extraction	Extraction
**Endosseous Neoformation**	0 No	/	/	/
	1 Non-odontogenic and included	Strict control	Strict control	Strict control
	2 Non-odontogenic but exposure risk	Surgery	Surgery	Surgery
	3 Odontogenic	Surgery	Surgery	Surgery
**Included Elements**	0 No, total endosseous inclusion with no contact with other elements	/	/	/
	1 Semi-included without communication with oral cavity	/	/	/
	2 Dysodontiasis without infection	/	/	Extraction
	3 Dysodontiasis with infection	Extraction	Extraction	Extraction
**Orthodontic Treatment**	0 No, or non-invasive treatment (traditional removable or fixed appliance, invisaligne)	/	/	/
	3 Invasive treatment (mini screws and bone anchors)	Removal	Removal	Removal

(to get the dental score, select the highest rating achieved). Treatment modality needs to be individualized based on oncological disease, and the patient’s condition.

**Table 3:** Medication-related osteonecrosis of the jaw (MRONJ) risk factors and predisposing conditions.

**Local factors-related**	Anatomic factors
	Poor oral hygiene
	Previous MRONJ
	Previous radiotherapy in the head and neck region
**Demographic and systemic factors and other medication factors**	Age
	Tobacco
	Anemia
	Diabetes
	Antiresorptive for osteoporosis < 3 years
	Antiresorptive for osteoporosis > 3 years
	Antiangiogenics + antiresorptives
	Corticosteroid therapy
	Rheumatoid arthritis
	Kind of tumor
**Genetic factors**	Single nucleotide polymorphisms (SNPs) in gene responsible for bone turnover

## Data Availability

Data will be available at the Department of Oral and Maxillo-Facial Sciences (Sapienza University of Rome).

## Appendix A

The following clinical folder was used by the dentists in the Head and Neck Departement of University of Rome “Sapienza” for patients who had to undergo bone therapy. The folder is divided into sections, follow the instructions to fill in.

Risk factors for MRONJ (tick Y for yes if the patient presents the risk factor, N if not. If at least one Y is marked, the risk factor score is 1).

**Local Factors-Related**	Anatomic factors		
	Poor oral hygiene		
	Previous MRONJ		
	Previous radiotherapy in the head and neck region		
**Demographic and Systemic Factors and Other Medication Factors**	Age		
	Tobacco		
	Anemia		
	Diabetes		
	Antiresorptive for osteoporosis < 3 years		
	Antiresorptive for osteoporosis > 3 years		
	Antiangiogenics+antiresorptives		
	Corticosteroid therapy		
	Rheumatoid arthritis		
	Kind of tumor		
**Genetic Factors**	Single Nucleotide Polymorphisms (SNPs) in gene responsible for bone turnover		
		**Y**	**N**

Dental formula at first visit Date …/…/… dental based score (See Table 2 and complete). (To get the dental score select the higher rating achieved).

**Dental Based Score at Basline**	**Denture**	**Decay**	**Root**	**Periodontal**	**Periimplant**	**Endosseous**	**Included**	**Orthodontic**
Score								
Total Score								

Total score at first visit (the total score at first visit is obtained by summing all the achieved scores):

_____ (oncological status at baseline) +__ (dental based score) +__ (pharmacological related score) +__ (risk factors) = _________

Clearance to oncological bone therapy: _____ (DATE: __/__/____)

First re-evaluation and recall program

First re-evaluation, 30 days after dental treatment: DATE:___/___/____

Oncological status: _______________

Pharmacological related score: ______________

New dental formula at recall visit Date …/…/….. (See Table 2).

**New Dental Score**	**Denture**	**Decay**	**Root**	**Periodontal**	**Periimplant**	**Endosseous**	**Included**	**Orthodontic**
score								
total score								
To get the dental score, select the highting rating achieved								

Total score at first recall:

_____(oncological status at baseline) + __ (new dental score)+ __ (farmacological related score) +__ (risk factors) = ___________ + __________

Enter the patient in the recall visits program_____________.

**Total Score**	**Recall**
1	Every 6 months
2	Every 4 months
3	Every 3 months
4	Every 2 months
5 or more	Every 1 months

Follow-up scores.

**Date**	**Onco** **Status**	**Dental Score**	**Pharma** **Score**	**Risk Factor**	**Final Score**	**Treatment**
						
						
						
						
						
						
						
						
						
						
						

## References

[1] Marx R.E. (2003). Pamidronate (Aredia) and zoledronate (Zometa) induced avascular necrosis of the jaws: A growing epidemic. J. Oral Maxillofac. Surg..

[2] Campisi G., Mauceri R., Bertoldo F., Bettini G., Biasotto M., Colella G., Consolo U., Di Fede O., Favia G., Fusco V. (2020). Medication-Related Osteonecrosis of Jaws (MRONJ) Prevention and Diagnosis: Italian Consensus Update 2020. Int. J. Environ. Res. Public Health.

[3] Drake M.T., Clarke B.L., Khosla S. (2008). Bisphosphonates: Mechanism of Action and Role in Clinical Practice. Mayo Clin. Proc..

[4] Baron R., Ferrari S.L., Russell R.G.G. (2011). Denosumab and bisphosphonates: Different mechanisms of action and effects. Bone.

[5] Christodoulou C., Pervena A., Klouvas G., Galani E., Falagas M.E., Tsakalos G., Visvikis A., Nikolakopoulou A., Acholos V., Karapanagiotidis G. (2009). Combination of Bisphosphonates and Antiangiogenic Factors Induces Osteonecrosis of the Jaw More Frequently than Bisphosphonates Alone. Oncology.

[6] Ruggiero S.L., Dodson T.B., Fantasia J., Goodday R., Aghaloo T., Mehrotra B., O’Ryan F. (2014). American Association of Oral and Maxillofacial Surgeons Position Paper on Medication-Related Osteonecrosis of the Jaw—2014 Update. J. Oral Maxillofac. Surg..

[7] Bedogni A., Fusco V., Agrillo A., Campisi G. (2012). Learning from experience. Proposal of a refined definition and staging system for bisphosphonate-related osteonecrosis of the jaw (BRONJ). Oral Dis..

[8] Bedogni A., Fedele S., Bedogni G., Scoletta M., Favia G., Colella G., Agrillo A., Bettini G., Di Fede O., Oteri G. (2014). Staging of osteonecrosis of the jaw requires computed tomography for accurate definition of the extent of bony disease. Br. J. Oral Maxillofac. Surg..

[9] Fedele S., Bedogni G., Scoletta M., Favia G., Colella G., Agrillo A., Bettini G., Di Fede O., Oteri G., Fusco V. (2015). Up to a quarter of patients with osteonecrosis of the jaw associated with antiresorptive agents remain undiagnosed. Br. J. Oral Maxillofac. Surg..

[10] Tenore G., Nuvoli A., Mohsen A., Cassoni A., Battisti A., Terenzi V., Della Monaca M., Raponi I., Brauner E., De Felice F. (2020). Tobacco, Alcohol and Family History of Cancer as Risk Factors of Oral Squamous Cell Carcinoma: Case-Control Retrospective Study. Appl. Sci..

[11] Kuroshima S., Sasaki M., Sawase T. (2019). Medication-related osteonecrosis of the jaw: A literature review. J. Oral Biosci..

[12] Yarom N., Shapiro C.L., Peterson D.E., Van Poznak C.H., Bohlke K., Ruggiero S.L., Migliorati C.A., Khan A., Morrison A., Anderson H. (2019). Medication-Related Osteonecrosis of the Jaw: MASCC/ISOO/ASCO Clinical Practice Guideline. J. Clin. Oncol..

[13] Khan A.A., Morrison A., Cheung A., Hashem W., Compston J.E. (2016). Osteonecrosis of the jaw (ONJ): Diagnosis and management in 2015. Osteoporos. Int..

[14] Kajizono M., Sada H., Sugiura Y., Soga Y., Kitamura Y., Matsuoka J., Sendo T. (2015). Incidence and Risk Factors of Osteonecrosis of the Jaw in Advanced Cancer Patients after Treatment with Zoledronic Acid or Denosumab: A Retrospective Cohort Study. Biol. Pharm. Bull..

[15] De Felice F., De Vincentiis M., Valentini V., Musio D., Mezi S., Mele L.L., Della Monaca M., D’Aguanno V., Terenzi V., Di Brino M. (2017). Management of salivary gland malignant tumor: The Policlinico Umberto I, “Sapienza” University of Rome Head and Neck Unit clinical recommendations. Crit. Rev. Oncol..

[16] De Felice F., De Vincentiis M., Valentini V., Musio D., Mezi S., Mele L.L., Terenzi V., D’Aguanno V., Cassoni A., Di Brino M. (2017). Follow-up program in head and neck cancer. Crit. Rev. Oncol..

[17] Capocci M., Romeo U., Cocco F., Bignozzi I., Annibali S., Ottolenghi L. (2014). The “CROMa” Project: A Care Pathway for Clinical Management of Patients with Bisphosphonate Exposure. Int. J. Dent..

[18] Cassoni A., Brauner E., Pucci R., Terenzi V., Mangini N., Battisti A., Della Monaca M., Ciolfi A., Laudoni F., Di Carlo S. (2020). Head and Neck Osteosarcoma—The Ongoing Challenge about Reconstruction and Dental Rehabilitation. Cancers.

[19] Stopeck A., Fizazi K., Body J.-J., Brown J.J., Carducci M., Diel I.J.I., Fujiwara Y., Martín M., Paterson A.H.G., Tonkin K. (2016). Safety of long-term denosumab therapy: Results from the open label extension phase of two phase 3 studies in patients with metastatic breast and prostate cancer. Support. Care Cancer.

[20] Brauner E., Jamshir S., Guarino G., Ciolfi A., Valentini V., Pompa G., Di Carlo S. (2016). Pleomorphic adenoma rehabilitative treatment in growing up patient: A 20-years fol-low-up. Eur. Rev. Med. Pharmacol. Sci..

[21] Brauner E., Quarato A., De Angelis F., Pompa G., Jamshir S., Valentini V., Di Carlo S. (2017). Prosthetic rehabilitation involving the use of implants following a fibula free flap reconstruction in the treatment of Osteosarcoma of the maxilla: A case report. La Clin. Ter..

[22] Valentini V., Terenzi V., Cassoni A., Bosco S., Brauner E., Shahinas J., Pompa G. (2012). Giant Cell Lesion or Langerhans’ Cell Histiocytosis of the Mandible? A Case Report. Eur. J. Inflamm..

[23] Henry D.H., Costa L., Goldwasser F., Hirsh V., Hungria V., Prausova J., Scagliotti G.V., Sleeboom H., Spencer A., Vadhan-Raj S. (2011). Randomized, Double-Blind Study of Denosumab Versus Zoledronic Acid in the Treatment of Bone Metastases in Patients With Advanced Cancer (Excluding Breast and Prostate Cancer) or Multiple Myeloma. J. Clin. Oncol..

[24] Capocci M., Romeo U., Guerra F., Mannocci A., Tenore G., Annibali S., Ottolenghi L. (2017). Medication-related osteonecrosis of the jaws (MRONJ) and quality of life evaluation: A pilot study. La Clin. Ter..

[25] Hinchy N.V., Jayaprakash V., Rossitto R.A., Anders P.L., Korff K.C., Canallatos P., Sullivan M.A. (2013). Osteonecrosis of the jaw—Prevention and treatment strategies for oral health professionals. Oral Oncol..

[26] Saad F., Brown J.E., Van Poznak C., Ibrahim T., Stemmer S.M., Stopeck A.T., Diel I.J., Takahashi S., Shore N., Henry D.H. (2011). Incidence, risk factors, and outcomes of osteonecrosis of the jaw: Integrated analysis from three blinded active-controlled phase III trials in cancer patients with bone metastases. Ann. Oncol..

[27] Yamazaki T., Yamori M., Ishizaki T., Asai K., Goto K., Takahashi K., Nakayama T., Bessho K. (2012). Increased incidence of osteonecrosis of the jaw after tooth extraction in patients treated with bisphosphonates: A cohort study. Int. J. Oral Maxillofac. Surg..

[28] Cassoni A., Romeo U., Terenzi V., Della Monaca M., Zadeh O.R., Raponi I., Fadda M.T., Polimeni A., Valentini V. (2016). Adalimumab: Another Medication Related to Osteonecrosis of the Jaws?. Case Rep. Dent..

[29] Brauner E., Valentini V., Jamshir S., Guarino G., Battisti A., Fadda M.T., Pompa G. (2016). Retrospective review of 78 rehabilitated head and neck postoncological patients: A new classification method. Minerva Stomatol..

[30] Pompa G., Saccucci M., Di Carlo G., Brauner E., Valentini V., Di Carlo S., Gentile T., Guarino G., Polimeni A. (2015). Survival of dental implants in patients with oral cancer treated by surgery and radiotherapy: A retrospective study. BMC Oral Health.

[31] Coleman R. (2001). Metastatic bone disease: Clinical features, pathophysiology and treatment strategies. Cancer Treat. Rev..

[32] Papi P., Brauner E., Di Carlo S., Musio D., Tombolini M., De Angelis F., Valentini V., Tombolini V., Polimeni A., Pompa G. (2019). Crestal bone loss around dental implants placed in head and neck cancer patients treated with different radiotherapy techniques: A prospective cohort study. Int. J. Oral Maxillofac. Surg..

[33] Cassoni A., Valentini V., Della Monaca M., Pagnoni M., Prucher G., Brauner E., Guarino G., Fadda M., Jamshir S., Pompa G. (2014). Keratocystic Odontogenic Tumor Surgical Management: Retrospective Analysis on 77 Patients. Eur. J. Inflamm..

[34] Brauner E., Valentini V., Guarino G., Cassoni A., Jamshir S., Minasi R., Fadda M., Pagnoni M., Pompa G. (2013). Osteoradionecrosis of a Mandible: A Case Report of Implant-Supported Rehabilitation. Eur. J. Inflamm..

[35] Brauner E., Pompa G., Quarato A., Jamshir S., De Angelis F., Di Carlo S., Valentini V. (2017). Maxillofacial Prosthesis in Dentofacial Traumas: A Retrospective Clinical Study and Introduction of New Classification Method. BioMed Res. Int..

[36] Romeo U., Galanakis A., Marias C., Del Vecchio A., Tenore G., Palaia G., Vescovi P., Polimeni A. (2011). Observation of Pain Control in Patients with Bisphosphonate-Induced Osteonecrosis Using Low Level Laser Therapy: Preliminary Results. Photomed. Laser Surg..

[37] Tenore G., Mohsen A., Rossi A.F., Palaia G., Rocchetti F., Cassoni A., Valentini V., Ottolenghi L., Polimeni A., Romeo U. (2020). Does Medication-Related Osteonecrosis of the Jaw Influence the Quality of Life of Cancer Patients?. Biomedicines.

[38] Di Carlo S., De Angelis F., Ciolfi A., Quarato A., Piccoli L., Pompa G., Brauner E. (2019). Timing for implant placement in patients treated with radiotherapy of head and neck. La Clin. Ter..

[39] Pompa G., Brauner E., Jamshir S., De Angelis F., Giardino R., Di Carlo S. (2017). Quality of life in patients rehabilitated with palatal obturator without reconstruction versus fixed implant-prosthesis after reconstruction of maxillectomy defects. J. Int. Dent. Med. Res..

